# Automated DNA extraction using cellulose magnetic beads can improve *EGFR* point mutation detection with liquid biopsy by efficiently recovering short and long DNA fragments

**DOI:** 10.18632/oncotarget.25388

**Published:** 2018-05-18

**Authors:** Chiho Nakashima, Akemi Sato, Tomonori Abe, Junichi Kato, Mitsuharu Hirai, Tomomi Nakamura, Kazutoshi Komiya, Eisaburo Sueoka, Shinya Kimura, Naoko Sueoka-Aragane

**Affiliations:** ^1^ Division of Hematology, Respiratory Medicine and Oncology, Department of Internal Medicine, Faculty of Medicine, Saga University, Saga, Japan; ^2^ Department of Clinical Laboratory Medicine, Faculty of Medicine, Saga University, Saga, Japan; ^3^ ARKRAY, Inc., Kyoto, Japan

**Keywords:** liquid biopsy, EGFR mutation, DNA extraction, pre-analytical procedure, plasma DNA integrity

## Abstract

The clinical utility of plasma DNA for detecting cancer-specific mutations has rapidly achieved recognition, but reliability has not been established because of relatively low mutation-detection rates compared with those from tissue re-biopsy. To address this shortcoming we examined efficiency, in terms of mutation detection, of an automated DNA extraction system that uses cellulose magnetic beads. A fully automated, highly sensitive point-mutation-detection method, mutation-biased PCR and quenching probe (MBP-QP) system, was used for this study. Plasma DNA was extracted from 61 plasma samples collected from patients with advanced non-small cell lung cancer. Extraction was performed manually with 200 μl plasma (200-M) by using a silica membrane spin column system or an automated system using 200 μl (200-A) or 1000 μl (1000-A) plasma. Median DNA yield quantified by real-time PCR was 4.4, 4.5, and 17.3 ng with the three methods, respectively. Sensitivity for detecting epidermal growth factor receptor (*EGFR*) L858R point mutation was 36.6%, 58.5%, and 77.5%, and specificity was 93.3%, 100%, and 96.7%, respectively. Concordance rates were 60.6%, 76.1%, and 85.7%. The size distribution of plasma DNA with automated extraction was bimodal with modes at about 170 bp and 5 Kb, and plasma DNA of both sizes included tumor-derived DNA. In this report, we demonstrate that automated DNA extraction using cellulose magnetic beads can improve mutation-detection rates with plasma DNA in association with two overall sizes of DNA fragments recovered by this DNA isolation system. Examining the biological characteristics of these fragments will be the subject of further investigation.

## INTRODUCTION

Clarifying the roles of oncogenes and their mutations can aid in the selection of individualized approaches to cancer treatment, including molecular targeted therapy. In patients with non-small cell lung cancer (NSCLC) harboring epithelial growth factor receptor (*EGFR*) activating/sensitive mutations, such as L858R and exon 19 deletions, EGFR tyrosine kinase inhibitors (EGFR-TKIs) have been highly effective in approximately 70% of cases [1, 2]. Despite this encouraging initial response, the vast majority of patients treated with 1st generation EGFR-TKIs acquire resistance about one year after treatment [2]. Approximately half of the cases of this acquired resistance are caused by a secondary *EGFR* mutation, T790M [3, 4]. Osimertinib is a 3rd generation EGFR-TKI that has a strong anti-cancer effect against lung cancers harboring T790M [5, 6]. In such cases, monitoring the dominant genetic alterations is invaluable for selecting optimal therapeutic agents. However, performing invasive tissue re-biopsy of primary or metastatic tumors is not always practicable.

As a result, interest in plasma DNA analysis has been growing. An increased level of double-stranded DNA fragments carrying tumor-specific genetic alterations is frequently observed in the blood of cancer patients [7–9]. Peripheral blood sampling is minimally invasive, so plasma DNA analysis can be performed repeatedly at any stage of disease and can reflect the entire range of molecular-biological characteristics of the tumor (including metastatic lesions), whereas the results from only one biopsied site cannot. Therefore, such “liquid biopsy” is considered the most suitable approach for serial monitoring of second- or third-site mutations [7]. The ESMO Clinical Practice Guidelines for diagnosis and follow-up of metastatic NSCLC propose liquid biopsy as a surrogate source of tumor DNA and a new strategy for tumor genotyping, mainly at the time of progressive disease (PD) in patients with tumors having *EGFR* mutations [10].

Because of the extremely small quantity of target mutations coupled with a high normal background level, the analysis of plasma DNA requires a highly sensitive detection system [11, 12]. Several methodologies, including droplet digital PCR (ddPCR), beads, emulsion, amplification and magnetics (BEAMing), cycleave PCR, and next generation sequencing, have been used for mutation detection [13–17]. As an alternative, we developed a fully-automated, highly sensitive mutation detection system named the mutation-biased PCR and quenching probe system (MBP-QP) [17]. The detection limit of MBP-QP for *EGFR* mutation is 0.3%, and a multicenter retrospective study revealed that T790M was detected in 53% of patients who acquired resistance to 1st EGFR-TKIs [18]. In a prospective, multicenter, observational study, T790M was observed in 40% of cases of NSCLC with PD after treatment with EGFR-TKIs, and 26% of them were positive for T790M before PD [7].

However, from the viewpoint of clinical application and the requirement to accumulate credible clinical data, pre-analytical procedures for liquid biopsy, such as blood collection, storage, and DNA extraction method—all of which can influence the analysis results—must be optimized and unified across laboratories [19–22]. In recent years, fully-automated DNA extraction systems have been developed. This equipment adopts cellulose magnetic beads for DNA capturing, and it might allow us to extract pure DNA easily with a capacity that is higher than that of a manual silica membrane spin column system. However, the technique has seldom been applied to plasma DNA, and its results with plasma DNA have not been evaluated.

In this study, we compared plasma DNA yields obtained by manual silica membrane spin columns with those from an automated DNA extraction system using cellulose magnetic beads. In addition, we compared the two DNA-extraction procedures in terms of tissue and plasma *EGFR* mutation detection sensitivity, specificity, and concordance (proportion of cases in which the result of tissue and plasma T790M tests were in agreement). We also investigated the DNA size distribution with each DNA extraction procedure. The purpose was to identify the optimum method of plasma DNA extraction for liquid biopsy.

## RESULTS

### Comparison of plasma DNA concentration depending on three different procedures for DNA isolation

**Figure 1:**
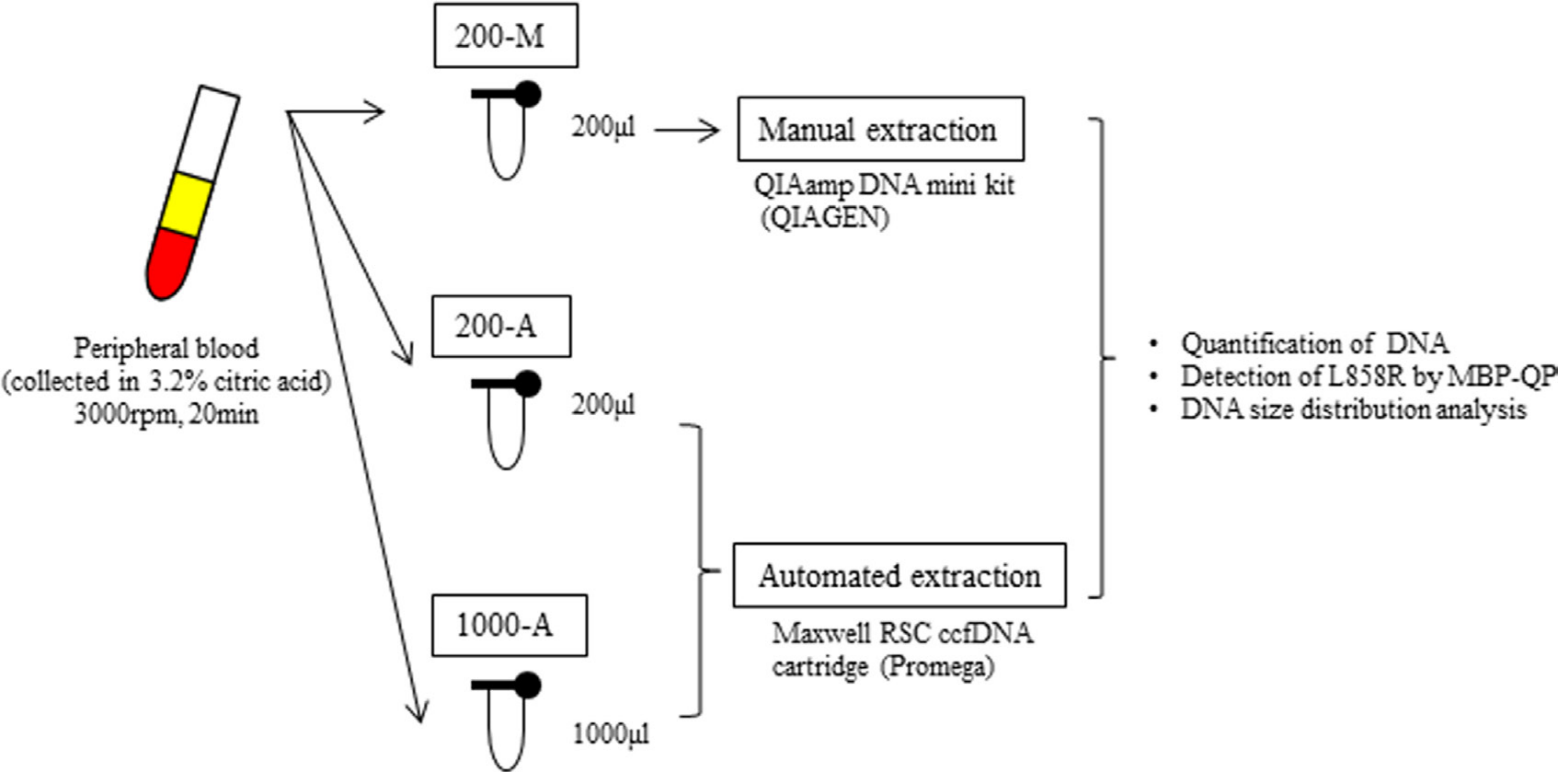
The design of this study We collected peripheral blood into tubes containing 3.2% citric acid. Blood was obtained from patients with advanced NSCLC (*N* = 61) and healthy volunteers (*N* = 10). Among 61 patients, 41 were verified to harbor *EGFR* L858R from tissue, and 20 were verified to not carry. After collection, blood samples were immediately centrifuged and the plasma from each tube was separated into two 200 μl aliquots and one 1000 μl aliquot. One 200 μl aliquot was subjected to manual plasma DNA extraction (200-M), the other 200 μl aliquot to automated extraction (200-A), and the 1000 μl aliquot to automated extraction (1000-A). The plasma volume for manual extraction was fixed by the limit of capacity with the QIAamp DNA mini Kit (QIAGEN).

The design of our study is illustrated in Figure 1. Plasma collected from 61 patients with lung cancer and 10 healthy volunteers was divided into three different DNA extraction groups: 200-M, 200-A, and 1000-A. 200-M refers to DNA isolated with manual extraction by a QIAamp DNA mini kit from 200 μl plasma. 200-A and 1000-A refer to DNA isolated with automated extraction by a Maxwell RSC ccfDNA plasma cartridge from 200 μl and 1000 μl plasma, respectively. Patient characteristics are shown in Supplementary Table 1. *EGFR* L858R was verified to be present in 41 patients and absent in 20 patients by using tissue specimens. Proportions of females were 80% among L858R-positive patients and 35% among L858R-negative patients. The percentage of never smokers was 66% among L858R-positive patients and 30% among L858R-negative patients. All patients were at an advanced stage of NSCLC (stage IV) or had metastatic disease recurrence.

We first compared plasma DNA yield of patients with advanced NSCLC to that of healthy volunteers using method 1000-A. Plasma DNA was measured by three different assays to determine the optimal measurement method (Supplementary Figure 1). Median plasma DNA concentration measured by qPCR was 20.1 ng/mL in patients and 3.3 ng/mL plasma in healthy individuals (*p* < 0.01; Supplementary Figure 1A). A similar result was obtained with Quantus, the fluorescent measurement of dsDNA intercalated dye (*p* < 0.01), and the results of these two methods were correlated (Supplementary Figures 1B, D). These data were consistent with previous reports [11, 23]. However, no difference was seen with NanoDrop, measured by UV absorbance at 260 nm (Supplementary Figure 1C). Thus, we concluded that the UV measurement system is not suitable for plasma DNA quantification because single-stranded DNA and RNA are included in the result.

**Figure 2:**
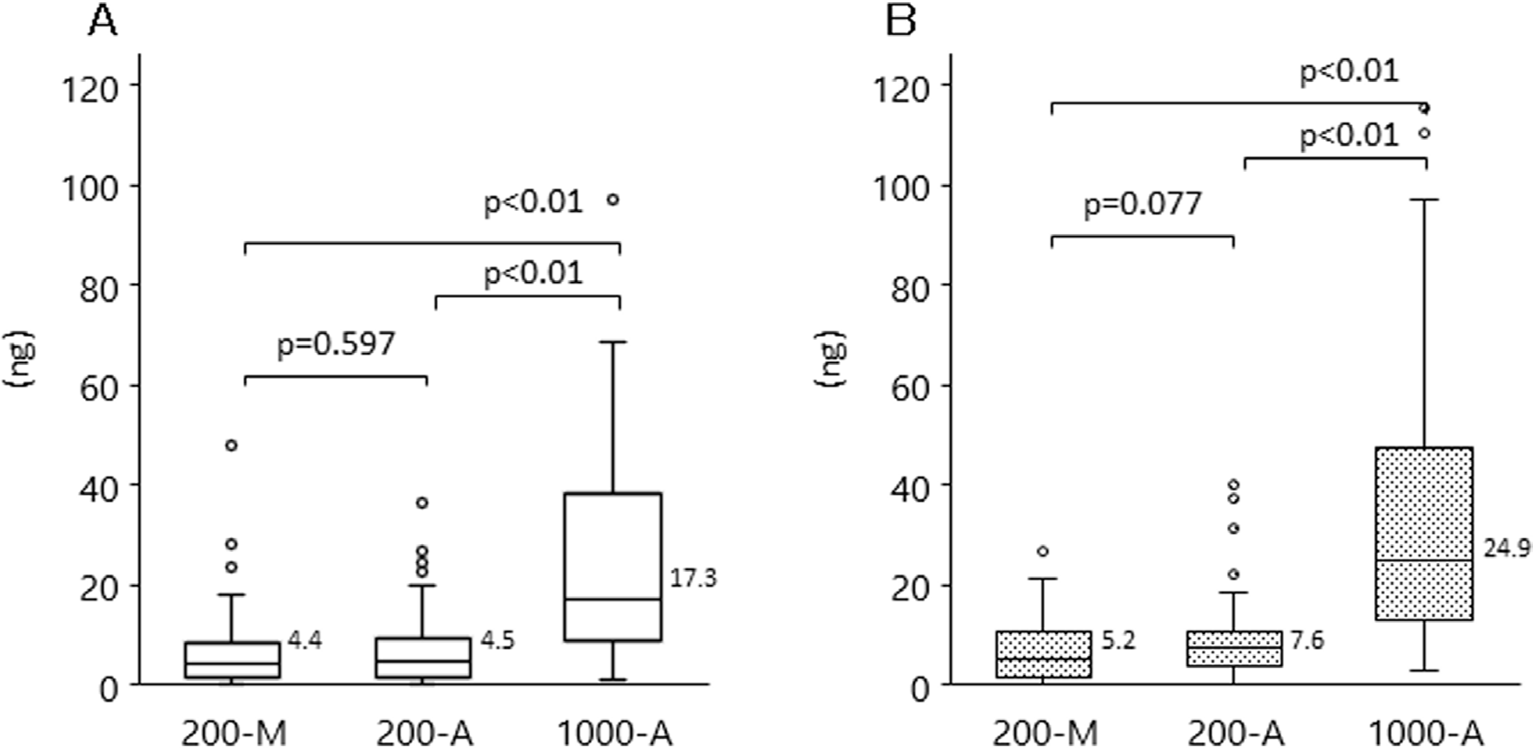
DNA yield with each extraction methods among patients with advanced NSCLC and healthy volunteers Plasma DNA was isolated by manual extraction (200-M) and automated extraction (1000-A). The concentration of plasma DNA was evaluated with qPCR (**A**), or Quantus, the fluorescent measurement of dsDNA intercalated dye (**B**). The *p*-value for the comparison between 200-M and 200-A is *p* = 0.597 (qPCR), *p* = 0.077 (Quantus); that between 200-M and 1000-A, and between 200-A and 1000-A, is *p* < 0.001 (both methods).

Next, we compared plasma DNA yield from the different DNA extraction methods (200-M, 200-A, and 1000-A) by using qPCR (Figure 2A) and Quantus (Figure 2B). The median plasma DNA yields with 200-M, 200-A, and 1000-A, when quantified by qPCR, were 4.4, 4.5, and 17.3 ng, respectively. With 1000-A, median plasma DNA yields measured by either qPCR or Quantus were approximately 4-fold higher than those with 200-M and 200-A. There was no difference between the DNA yields measured with 200-M and 200-A.

### Detection of L858R was improved by automated plasma DNA extraction

**Figure 3:**
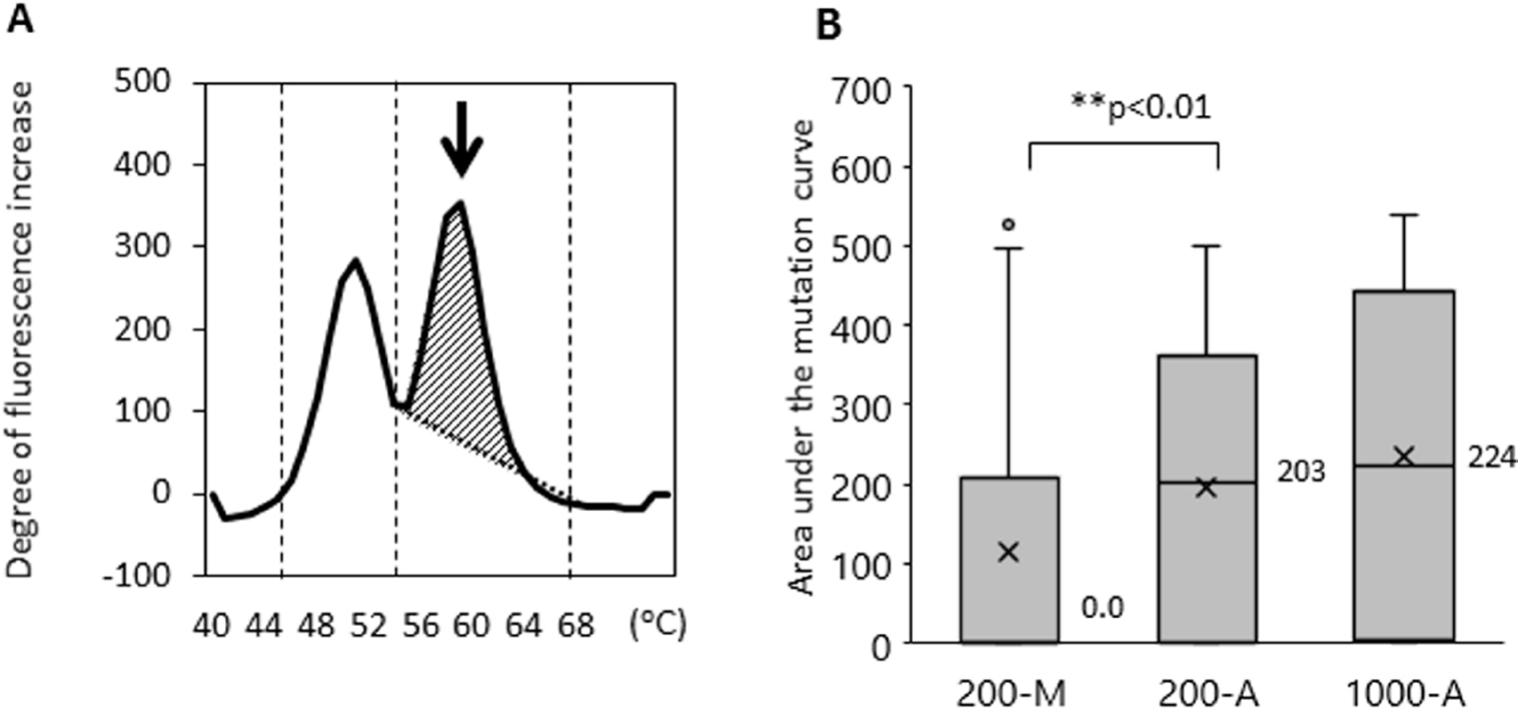
The area under the mutant curves (mutant AUC) was correlated with the mutant allele copy number The mutant AUC is illustrated in (**A**). The arrow indicates the mutation peak. The mutant AUC for L858R was defined as the integral under the curve between 56° C and 68° C using the MBP-QP method, and was calculated by the *i*-densy AreaAna^®^ software. (**B**) The mutant AUC with plasma DNA extracted by the automated method (200-A, 1000-A) and that by manual procedure (200-M). The numbers next to the bar charts are the mean values of mutant AUC, and an X represents the average of mutant AUC. Freidman's test was used for analysis.

We then examined whether the method of DNA extraction influences the efficiency of *EGFR* L858R mutation detection. The limit of *EGFR* L858R mutation detection using the updated MBP-QP method was 2 copies per test using control plasmid, 0.01 ng per test using H1975 cell line DNA, and 0.1% allele frequency (AF) determined with genomic DNA from a mixture of H1975 and A549 cells (Supplementary Figure 2). Using this upgraded highly sensitivity system, we examined L858R with patients’ plasma DNA isolated by the 200-M, 200-A, and 1000-A methods (Table 1). In terms of *EGFR* L858R point mutation detection, sensitivity was 36.6% (200-M), 58.5% (200-A), and 77.5% (1000-A); specificity was 93.3% (200-M), 100% (200-A), and 96.7% (1000-A); and concordance was 60.6% (200-M), 76.1% (200-A), and 85.7% (1000-A). Although specificity did not differ substantially among the methods, sensitivity and concordance were significantly better with the automated DNA extraction system. As we have already reported, mutant allele copy number is correlated with area under the curve of the mutant peak (mutant AUC) [24], which was defined by integrating the curve between 55–68° C for L858R using the MBP-QP method. The median values of mutant AUC were 0.0, 203, and 224 with 200-M, 200-A, and 1000-A, respectively (Figure 3). Although the amount of plasma DNA itself did not differ to a practical extent between the 200-M and 200-A methods (as mentioned in the previous section), the median value of mutant AUC with 200-A was significantly higher than with 200-M (*p* < 0.01).

**Table 1:** Comparison of L858R detection on different plasma DNA isolation systems

		PlasmaDNA						
		200-M		200-A		1000-A		
		L858R Positive	L858R Negative	L858R Positive	L858R Negative	L858R Positive	L858R Negative	
**Tissue**	L858R Positive (*n* = 41)	15	26	24	17	31	9	
	L858R Negative (*n* = 30)	2	28	0	30	1	29	
**Total (*n* = 71)**		17	51	24	47	32	38	
**Sensitivity**		36.6%		58.8%		77.5%		^**^P < 0.01
**Specificity**		93.3%		100%		96.7%		*P* = 0.36
**Concordance**		60.6%		76.1%		85.7%		^**^P < 0.01

### Plasma DNA size distribution demonstrated two peaks and the automated DNA extraction system can efficiently extract long DNA fragments

**Figure 4:**
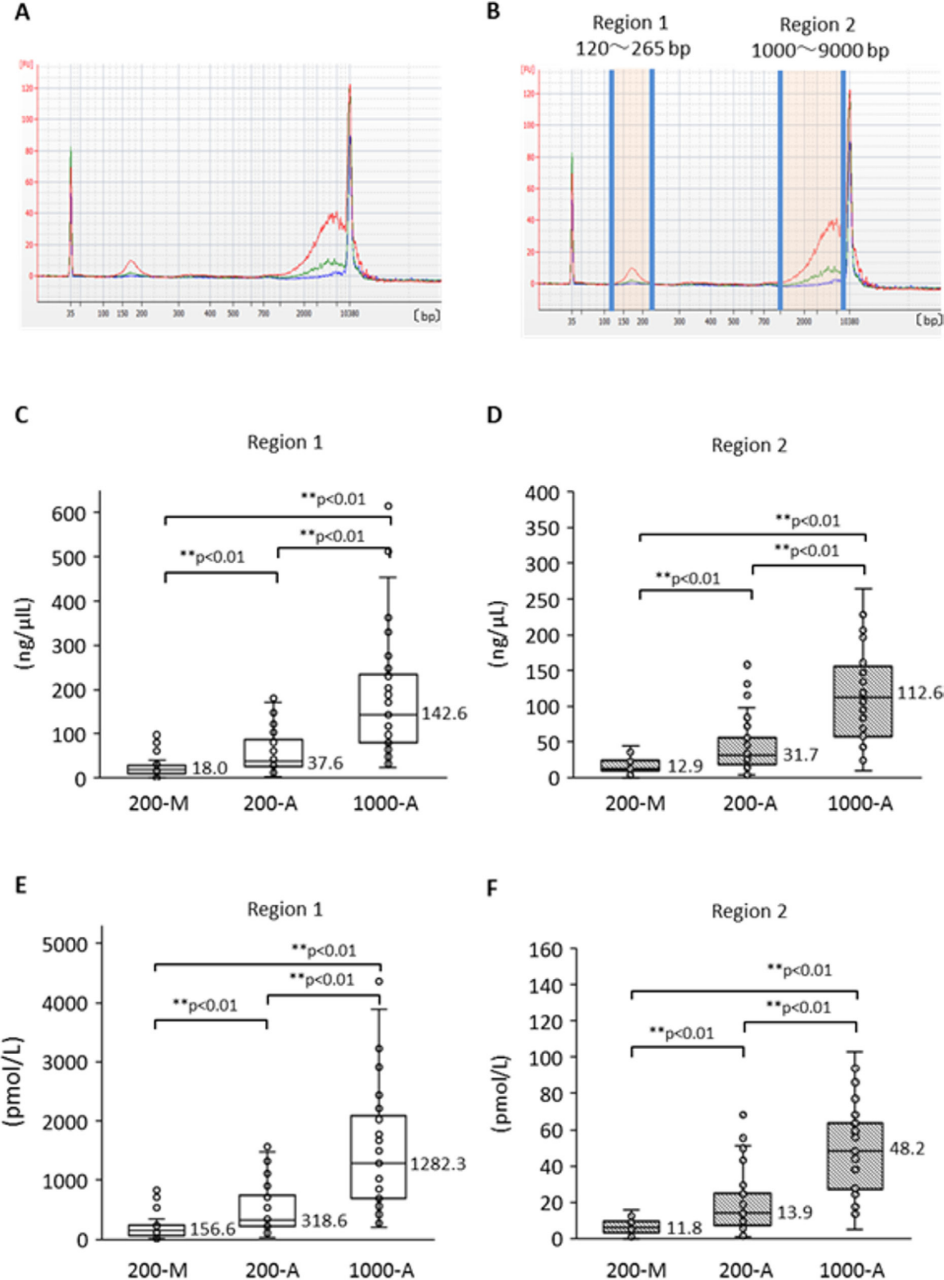
Size distribution of plasma DNA analyzed by Agilent Bioanalyzer and its difference according to extraction method (**A**) Representative size distribution pattern with each plasma DNA extraction method. Blue shows 200-M, green is 200-A, and red is 1000-A. (**B**) The definitions of “Region 1” and “Region 2”. DNA concentration and molality were measured by an Agilent Bioanalyzer. Comparison among DNA isolation procedures (200-M, 200-A, and 1000-A) was performed for concentration (**C**, **D**) and molarity (**E**, **F**) with Freidman's test and multiple pairwise comparisons.

To compare the quality of plasma DNA extracted manually with that extracted automatically, we investigated the plasma DNA size distribution by capillary electrophoresis. We first made a comparison among the DNA extraction methods: 200-M, 200-A, and 1000-A. Plasma DNA size-distribution patterns from a representative case of advanced NSCLC are shown in Figure 4A according to extraction method. The 1000-A method (red line) surpasses the other methods over the entire range. Furthermore, despite the 200-A and 200-M methods being based on the same original plasma volume, the DNA amount with the 200-A method exceeds that of the 200-M method in two regions. We call these regions “region 1” and “region 2” (Figure 4B), and we compared them statistically; the DNA concentrations are shown in Figure 4C and 4D, and the molarity is shown in Figure 4E and 4F. Freidman's test produced *p* < 0.01, so we performed pairwise comparisons. The results show that 1) the A methods can extract both short and long fragments significantly more effectively than the M method, and 2) the 1000-A method can extract significantly more fragments of both sizes than can the 200-A method, as a result of greater plasma volume. These results suggest that the method using cellulose magnetic beads can extract a broad range of DNA sizes more efficiently than the method using a silica membrane spin column.

Next, we compared plasma DNA size pattern from patients with advanced NSCLC to that from healthy individuals. Supplementary Figure 3 shows results for five representative cases of advanced NSCLC (Supplementary Figure 3A) and samples from healthy individuals (Supplementary Figure 3B). All plasma DNA in this experiment was isolated by the 1000-A method. Plasma DNA from healthy individuals showed only a single peak at 170 bp (short fragments), which is the size of a single DNA nucleosome unit, whereas plasma DNA from patients with advanced NSCLC revealed bimodality with peaks in region 1 (around 170 bp) and region 2 (around 5 Kb). Both short and long DNA fragments were observed in plasma from NSCLC patients significantly more frequently than in plasma from healthy volunteers (Supplementary Figure 3C–3F).

**Figure 5:**
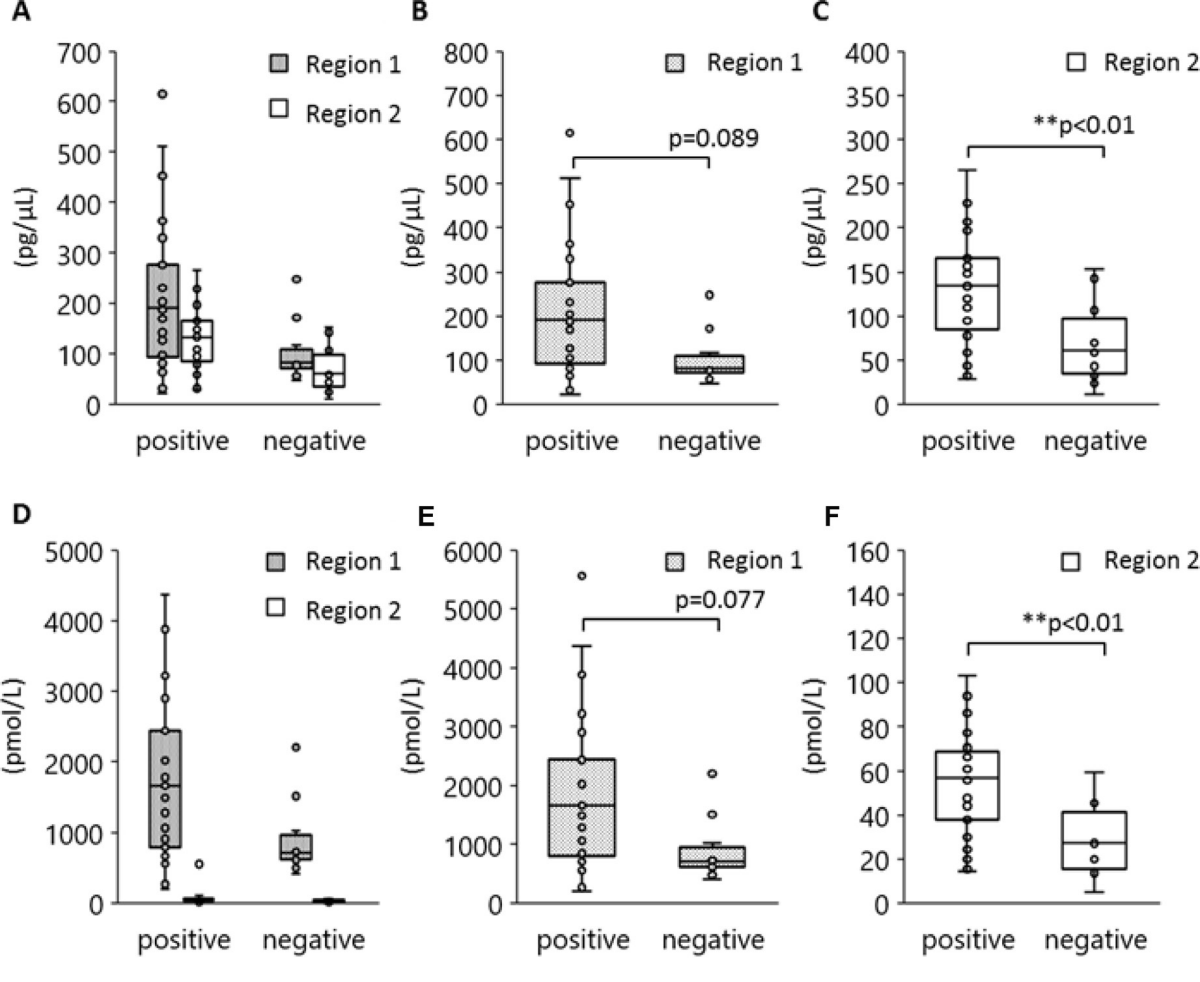
Association between *EGFR* L858R positivity and DNA amounts in regions 1 and 2 Peripheral blood was collected from 40 patients with NSCLC who carried *EGFR* L858R as verified by tissue biopsy and plasma DNA extracted by method 1000-A. DNA concentration and molarity were analyzed according to plasma L858R positivity as determined by the MPB-QP method. Concentration and molality of regions 1 and 2 DNA fragments were measured by an Agilent Bioanalyzer. The concentration (**A**–**C**) and molarity (**D**–**F**) of regions 1 and 2 are shown. Statistical analyses were performed with the Mann–Whitney *U* test. Two asterisks denote *p* < 0.01.

Although the 170 bp short fragments of plasma DNA are well known to be products of apoptosis, the origin of the 5 Kb long fragments is still unclear. Figure 5 shows the results of a statistical analysis to determine which size of plasma DNA contributes more towards L858R detection. Peripheral blood specimens were obtained from 40 patients with tumors harboring L858R as established by tissue biopsy. The plasma DNA was automatically extracted from 1000 μl plasma with a cellulose magnetic beads system (1000-A). We divided these specimens into two groups in terms of plasma L858R positivity determined by the MPB-QP method, and we measured plasma DNA integrity of these samples by capillary electrophoresis. The concentration (Figure 5A–5C) and the molarity (Figure 5D–5F) of region 1 and 2 fragments are also shown. Plasma specimens positive for L858R contained relatively higher concentration and molarity of both short and long fragments than L858R-negative plasma (Figure 5A, 5D). Comparisons between L858R-positive and L858R-negative specimens made separately for regions 1 and 2 revealed that long fragments were higher in concentration (Figure 5B, 5E) and molarity (Figure 5C, 5F) in L858R-positive than in L858R-negative specimens (*p* = 0.005 for concentration, *p* = 0.003 for molarity). These results imply that tumor-derived DNA might be more prevalent among long fragments in plasma DNA than among short fragments.

### L858R mutation was detected not only in short fragments but also in long fragments of plasma DNA

**Figure 6:**
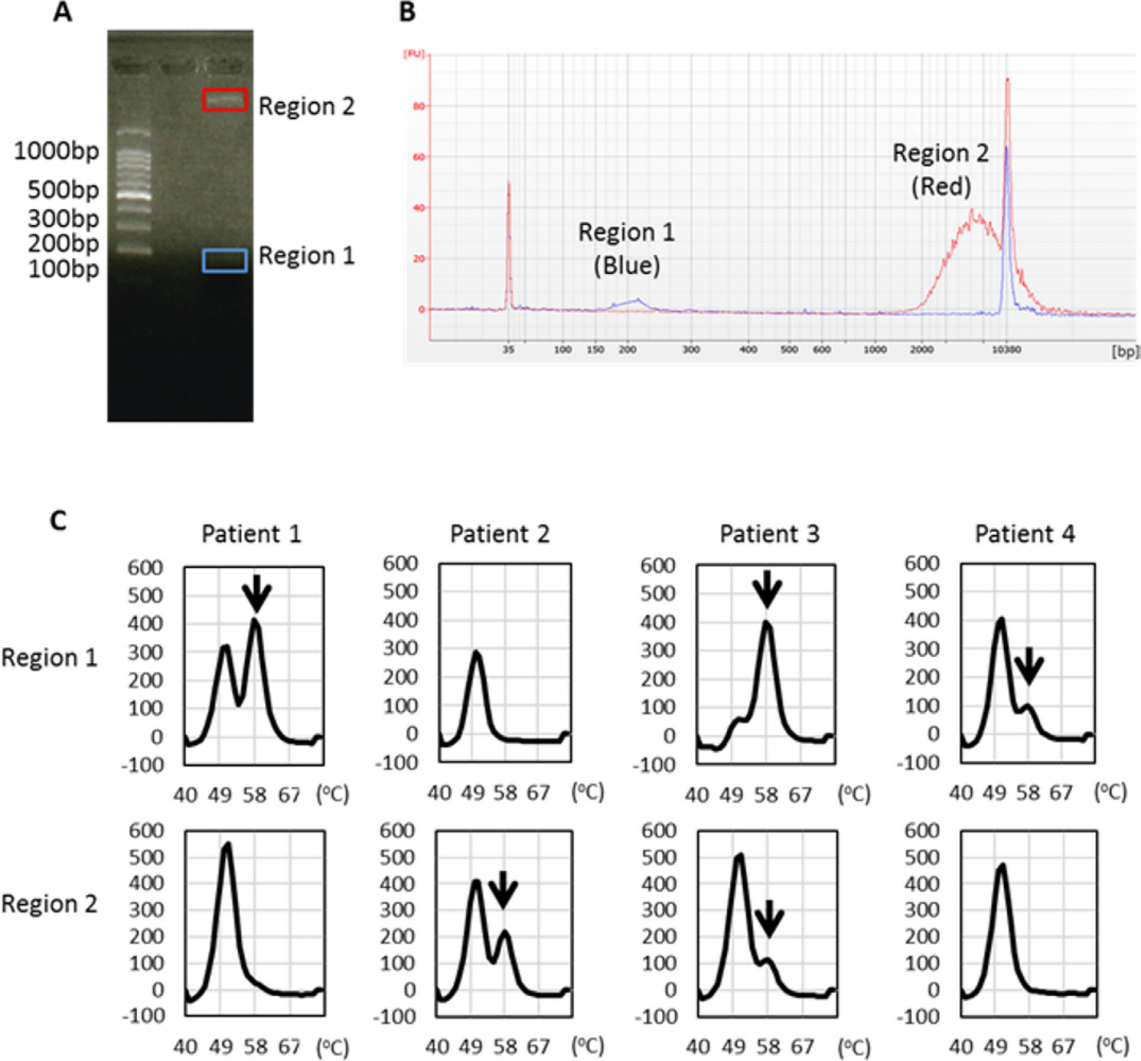
*EGFR* L858R detection from distinct sizes of plasma DNA isolated from regions 1 and 2 (**A**) Representative figure of agarose gel electrophoresis and locations of gel cutting. (**B**) Each targeted DNA size peak was identified by capillary electrophoresis. (**C**) *EGFR* L858R was detected in DNA eluents from regions 1 and 2 using the MBP-QP method. Arrows indicate the L858R peak.

To verify the importance of long fragments as containing tumor-derived DNA, we separated short and long plasma DNA fragments using agarose gel electrophoresis (Figure 6A) and examined the presence of L858R mutation by the MBP-QP method. We could identify by capillary electrophoresis the peak of each targeted size of DNA (Figure 6B) despite inevitable sample loss during these procedures. In two-fourths of the patients, we were able to detect L858R point mutation in region 2 DNA that matched the molarity of region 1 (Figure 6C). These results verify that not only short fragments but also long fragments of plasma DNA contained tumor-derived DNA.

## DISCUSSION

The utility of liquid biopsy has been highlighted over the past few years, and a number of highly sensitive mutation detection systems have been developed. Rapidly progressing innovative technologies, such as next generation sequencing, will doubtless be adapted to use for liquid biopsy, but an optimum pre-analytical procedure has not yet been established. In this report, we revealed that merely the DNA extraction method has a profound influence on the rate of detection of tumor-specific mutation with liquid biopsy, despite being based on the same peripheral blood specimen. Furthermore, the automated DNA extraction system using cellulose magnetic beads allows us to easily achieve a large quantity and a high quality of DNA yield and even improve the rate of detection of *EGFR* mutation L858R.

The DNA extraction procedure comprises several steps: 1) protein degeneration, 2) separation and capture of nucleic acid, 3) washing to exclude contaminants, and 4) elution. We have used silica membrane spin columns for these steps. However, this instrument has some problems, such as unavoidable sample loss, a limitation on capacity for plasma input, difficulty of elution with long DNA fragments, and being labor-intensive [25]. A newly automated system using cellulose magnetic beads for capturing DNA was recently developed and is now commercially available. Because a matrix secondary fibril-associated cellulose was applied to DNA purification in this system, a wide range of DNA sizes (from 100 bp to 50 Kb) could be isolated [26], leading to high DNA yield. In addition to isolating a large quantity of DNA, this system enabled us to improve the efficiency of *EGFR* mutation detection. Although the amount of plasma DNA itself did not differ significantly between the 200-M and 200-A methods, the sensitivity of L858R detection and the median value of area under the mutation peak, which implies mutant allele frequency, were significantly higher with 200-A than with 200-M.

From the point of view of DNA quality, we showed that the automated system allows us to observe peaks of two different sizes of plasma DNA. Furthermore, because L858R positivity was correlated with the amount of long fragment (5 Kb), we also showed that the long fragment contains tumor-derived DNA. Although tumor-derived DNA has been found in peripheral blood for over 40 years, the biological mechanisms of release have yet to be elucidated. Several mechanisms have been proposed: apoptosis, necrosis, and release of cells into the bloodstream followed by their lysis. In apoptotic cell death, inter-nucleosome cleavage of genomic DNA leads to a typical DNA fragmentation pattern called “apoptotic ladder”, which presents an integral multiple of 170 bp [27], whereas necrotic cell death causes the release of various sizes of DNA fragments that are much longer than those produced by apoptosis. In fact, many articles have reported that cancer patients present with increased plasma DNA integrity [28, 29]. Tumors experience both apoptotic and necrotic cell death, so our observation, that plasma DNA from patients with advanced NSCLC show a bimodal size distribution, is consistent. Thus, optimal DNA extraction methods would be expected to recover a broad range of DNA fragment sizes.

As for 170 bp DNA fragments, most likely the nucleosome unit, nucleosome-depleted region (NDR), was reduced in plasma (according to whole-genome sequencing of plasma DNA), and its frequency was correlated with mRNA expression level since NDR was its promoter region [30]. This result indicates that protein, histone in the case of the nucleosome, can protect against DNA degradation in the circulation. Looking back on our results, it is fascinating that such long DNA is maintained without enzymatic degradation (e.g. by DNase) within the circulation. Mechanisms surely exist that can maintain DNA without degradation. In cell culture, the size of extracellular DNA isolated from culture media was investigated, and large sized DNA, 2 Kb, which was assumed to form a complex with protein, was observed independently of cell cycle and the occurrence of apoptosis or necrosis [31, 32]. According to these results, the authors speculate that DNA was actively released from cells, but the phenomenon has not been observed in clinical samples. Although it remains unclear whether the long plasma DNA has some pathophysiological effect on cancer progression, tumors may utilize these long DNA molecules to achieve greater aggressive potential, drug resistance, or tumor-specific metabolism. Further examination of this problem should be made.

Considering the future clinical setting, because of its relative non-invasiveness, liquid biopsy will be an indispensable technique for monitoring drug response, drug resistance, and recurrence of disease [7, 33–35]. Enhancing the reliability of plasma DNA analysis will become increasingly important. Hence, we can suggest—as a result of the results presented in this report—that high-quality plasma DNA extracted with an automated DNA-extraction system is useful for analyses of plasma DNA, such as with a highly sensitive mutant-detection system.

## MATERIALS AND METHODS

### Cell lines

Human lung cancer cell lines H1975 (ATCC CRL-5905™) and A549 (ATCC CCL-185™) were purchased from American Type Culture Collection (Manassas, VA). Cells of A549, an *EGFR* wild type (WT) cell line, were cultured in RPMI-1640 medium supplemented with 10% fetal bovine serum at 37° C in 5% carbon dioxide. H1975 cells, which carry the *EGFR* L858R/T790M mutation, were cultured in RPMI-1640 containing high glucose, L-glutamine, and HEPES (ATCC #30-2001, Manassas, VA), and supplemented with 10% fetal bovine serum, at 37° C in 5% carbon dioxide.

### Patient selection

Peripheral blood specimens were obtained from 61 patients with advanced lung cancer and 10 healthy individuals. All cancer patients were treated and examined at a single center, Saga University Hospital, from February 2014 to April 2016, and the analysis was retrospective. Clinical stage was determined according to the current 7th edition of the TNM classification. Among the 61 patients, 41 had *EGFR* L858R mutation that was verified in tissue obtained by transbronchial lung biopsy or pleural effusion; verification was by the PNA-LNA PCR clamp assay (LSI Medience Corporation, Tokyo, Japan) or cycleave PCR technique (SRL Inc., Tokyo, Japan). As for the remaining 20 patients, that their tumor did not have *EGFR* mutations was verified by tissue biopsy or effusion. In previous studies by our group, we checked T790M to monitor the resistance to EGFR-TKIs. For monitoring drug resistance, T790M is superior to *EGFR* activating mutations, such as the L858R exon 19 deletion. However, to evaluate the effect of DNA extraction methods on the mutation detection rate, we considered that it is more appropriate to target L858R than T790M. There are three reasons for this. First, in plasma, detecting T790M is more difficult than L858R because of tumor heterogeneity. Even when T790M is detected in tissue, it is not uncommon for it not to be detected in peripheral blood, and vice versa. Second, detection of T790M is affected by treatment condition, tumor volume, and tumor heterogeneity because of its low allelic frequency. Third, to obtain large numbers of plasma positive for T790M-positive is more difficult than for L858R, because T790M is detected mainly in patients with resistance to EGFR-TKI. Therefore, to clarify our purpose, we consider that it is preferable to target L858R. Detection of exon 19 deletion is by the WIP-QP method, which is different from MBP-QP, so we did not examine exon 19 deletion in this study. The Clinical Research Ethics Committees of Saga University Hospital approved the study protocol. All patients gave informed consent for blood sample collection, and all testing was done according to the Declaration of Helsinki.

### DNA extraction methods

Peripheral blood specimens from the patients with lung cancer and healthy individuals were collected into tubes containing 3.2% citric acid. Immediately after blood sampling, specimens were put into a refrigerator and kept at 4° C until used. For further processing, specimens were centrifuged for 20 min at 3000 rpm, still at 4° C, and supernatants were collected and stored at –80° C. For manual DNA extraction (200-M), we isolated DNA from 200 μl plasma using a QIAamp DNA mini kit (QIAGEN, Hilden, Germany, Product no. 51304), and the DNA was eluted with 60 μl DNase-free water. For automated DNA extraction (200-A, 1000-A), we isolated DNA from 200 μl or 1000 μl plasma using a Maxwell RSC ccfDNA plasma cartridge (Promega, Mannheim, Germany, Product no. AS1480) according to the manufacturer's instructions. Each DNA sample extracted by Maxwell RSC was eluted with 60 μl TE buffer. Solutions containing extracted DNA were stored at –80° C until further processing.

### Quantification of plasma DNA

DNA concentrations were measured by UV absorbance at 260 nm using NanoDrop 2000C (Thermo Fisher Scientific Inc., Wilmington, USA), and the quantitative real-time PCR reaction (qPCR) was applied to *EGFR* exon 21 using StepOnePlus (Applied Biosystems, Darmstadt, Germany). The qPCR was performed initially at 95° C for 10 min, then in 40 cycles at 95° C for 15 s, and finally at 60° C for 60 s. The primer sets for qPCR were 5ʹ-AGGAACGTACTGGTGAAAAC ACCGC-3ʹ for the forward primer and 5ʹ-GCCTCCTTCT GCATGGTATTCTTTCTC -3ʹ for the reverse primer. In addition to these quantification methods, fluorescent dye intercalated with dsDNA was measured with Quantus (Promega, Mannheim, Germany). Briefly, Quantus constructs a standard curve of lambda DNA in each assay to facilitate comparison among assays. All of these experiments were done in triplicate.

### Evaluation of extracted DNA fragment size

The size distribution of plasma DNA was examined by a capillary electrophoresis system. We used the High Sensitivity DNA Kit (Agilent Technologies Inc., Santa Clara, CA, Product no. 5067–4626), a microchip, and analyzed it with the Agilent 2100 Bioanalyzer equipped with Expert 2100 software (Agilent Technologies Inc., Santa Clara, CA) according to the manufacturer's instructions. The concentration and molarity of each specified region was normalized by a ladder and by lower and upper markers. We defined “region 1” as plasma DNA of size 120~265 bp and “region 2” as 1000~9000 bp.

### The MBP-QP method for detection of *EGFR* L858R

The *EGFR* L858R mutation was detected by the MBP-QP method using *i*-densy™ IS 5320 (ARKRAY Inc., Kyoto, Japan). The MBP-QP method was developed to detect *EGFR* point mutations in plasma DNA, as we previously reported [17, 18]. To detect the L858R mutation, we have upgraded the L858R primer and probe. The new primer sets used were 5ʹ-TGGTGAAAACAC CGCATGTC-3ʹ for the forward primer, 5ʹ-ACACTACCCA GCAGTTTAGCCC-3ʹ for the reverse primer of the mutant sequence, and 5ʹ-CTGTACCAGCAGTATGGCCA-3ʹ for the reverse primer of the wild-type sequence. At the MBP step, PCR conditions were 95° C for 60 s, 50 cycles at 95° C for 1 s, and finally 60° C for 15 s. The PCR product size was 110 bp. The L858R mutation was detected at the QP step by the fluorescence intensity of a TAMRA-conjugated guanine-specific quenching fluorophore probe (QProbe, J-Bio21, Tokyo, Japan). This probe was designed complementary to L858R: 5ʹ-TTGGCCCGCCCAAAATC-(TAMRA)-3ʹ. The dissociation temperatures were 58° C for mutant and 51° C for wild type (Supplementary Figure 4).

The area under the mutant peaks was determined with the *i*-densy AreaAna software (ARKRAY Inc., Kyoto, Japan). A sample was declared positive for the L858R mutation with the MBP-QP method if the ratio of areas under mutation and wild-type peaks, multiplied by 100, was 1.3 or greater. We determined this criterion based on ± 3SD of the ratio of areas under mutation and wild-type peaks with control plasma DNA obtained from healthy volunteers. The control plasmids were prepared by GenScript USA Inc. as described previously [18].

### Separation of plasma DNA using agarose gel electrophoresis

We separated plasma DNA from each of the two peak sizes by using 2% low-melting agarose gel electrophoresis applied to the samples one-by-one. After electrophoresis, we blindly cut 200 mg gel pieces corresponding to two DNA sizes (100–200 bp, >1 Kb), and as a negative control we cut 200 mg gel pieces from a section of the same gel that did not include sample. The 100 bp DNA ladder was used as a reference for gel cutting.

From these gels, we extracted each size of plasma DNA using the NucleoSpin Gel and PCR Clean-up kit (Macherey-Nagel, Duren, Germany, Product no.740609.50), according to the manufacturer's Gel Clean-up instructions. Briefly, gel pieces were incubated at 50° C for 10 min with extraction buffer. The entire volume of each sample was added into a spin column and centrifuged at 11,000 g for 1 min at room temperature, then the columns were washed twice, and plasma DNA was eluded into 60 μl of elution buffer heated at 72° C. After drying the samples, we eluted them into 10 μl of nuclease-free water for the DNA size distribution analysis.

### Statistical analysis

Plasma DNA levels in lung cancer patients and healthy individuals were compared by the nonparametric Mann–Whitney *U* test for continuous variables. The DNA yields, concentration, and molarity of 170 bp and 5 Kb species from each DNA isolation procedure were compared by Freidman's nonparametric test for correlated outcomes, and the result, if statistically significant, was followed by multiple pairwise comparisons. Freidman's test was also used to compare areas under the mutation peaks from the three DNA extraction methods. The concentration and the molarity of regions 1 and 2 for each plasma *EGFR* L858R status were analyzed with the nonparametric Mann–Whitney *U* test. The chi-square test was used to compare sensitivity, specificity, and concordance for L858R detection depending on procedure for DNA isolation. *P* values are two-tailed; those below 0.01 were considered statistically significant. Analyses were conducted with SPSS version 19 (IBM SPSS Statistics, IBM, Tokyo, Japan).

## CONCLUSIONS

The choice of DNA extraction method has an enormous influence on efficiency of mutation detection in plasma DNA. For maximal detection of tumor-derived mutations, we recommend using the DNA extraction system based on cellulose magnetic beads with sufficient plasma volume.

## SUPPLEMENTARY MATERIALS FIGURES AND TABLES


