# Supplementary material for: Automated DNA extraction using cellulose magnetic beads can improve EGFR point mutation detection with liquid biopsy by efficiently recovering short and long DNA fragments
Source: Oncotarget. 2018 May 18;9(38):25181–92. doi: 10.18632/oncotarget.25388 (PMC5982773; doi:10.18632/oncotarget.25388)
Supplement: Supplementary file 1 [file oncotarget-09-25181-s001.pdf]

Automated DNA extraction using cellulose magnetic beads can improve *EGFR* point mutation detection with liquid biopsy by efficiently recovering short and long DNA fragments

SUPPLEMENTARY MATERIALS

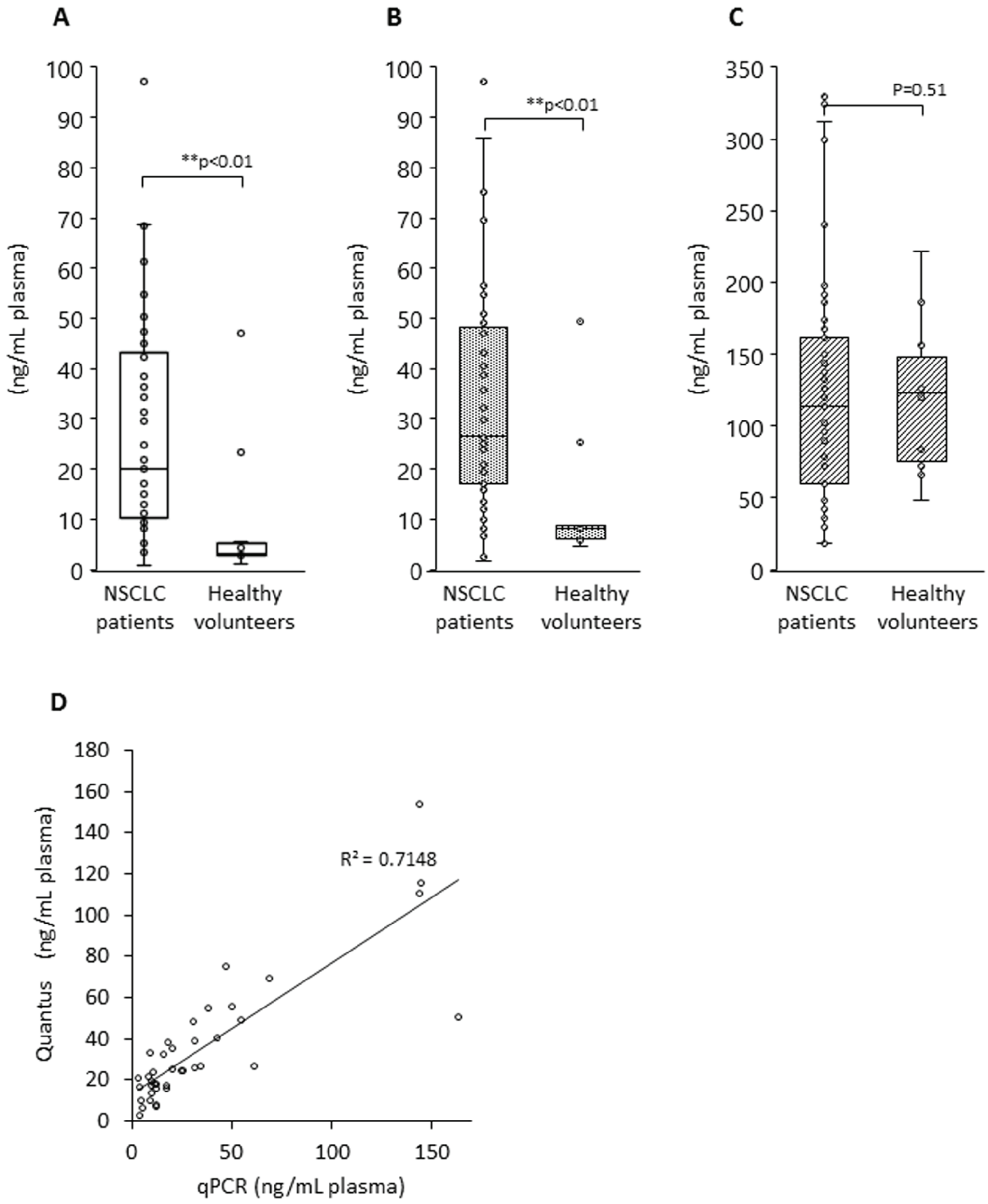

Supplementary Figure 1: Comparison of DNA yield between patients with advanced NSCLC and healthy volunteers. Plasma DNA was isolated by automated extraction (1000-A). Concentration of plasma DNA was evaluated with qPCR (**A**), or Quantus; the fluorescent measurement of dsDNA intercalated dye (**B**), and NanoDrop; UV absorbance at 260 nm (**C**). Statistical analyses were performed with the Mann–Whitney *U* test. The correlation of DNA yield measurement between qPCR and Quantus is shown in (**D**).

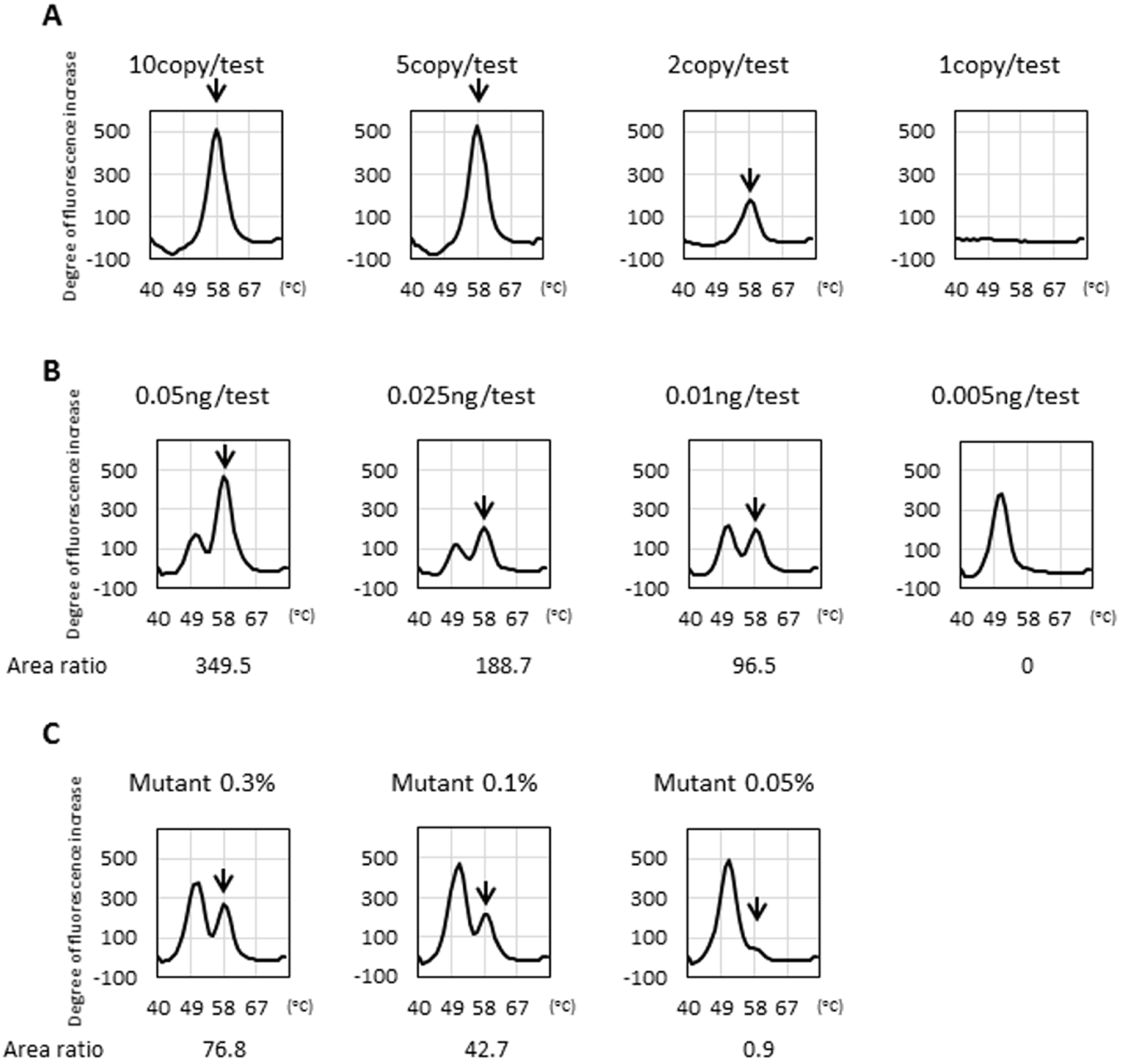

Supplementary Figure 2: Limit of detection for *EGFR* L858R by the MBP-QP method. The indicated copy number (**A**) or DNA amount (**B**) of control plasmid was applied to MBP-QP. Serial percentage of genomic DNA isolated from H1975 carrying L858R was mixed with that from A549 as wild type (**C**). The criterion of L858R positive is described in "PATIENTS AND METHODS". All assays were done in triplicate.

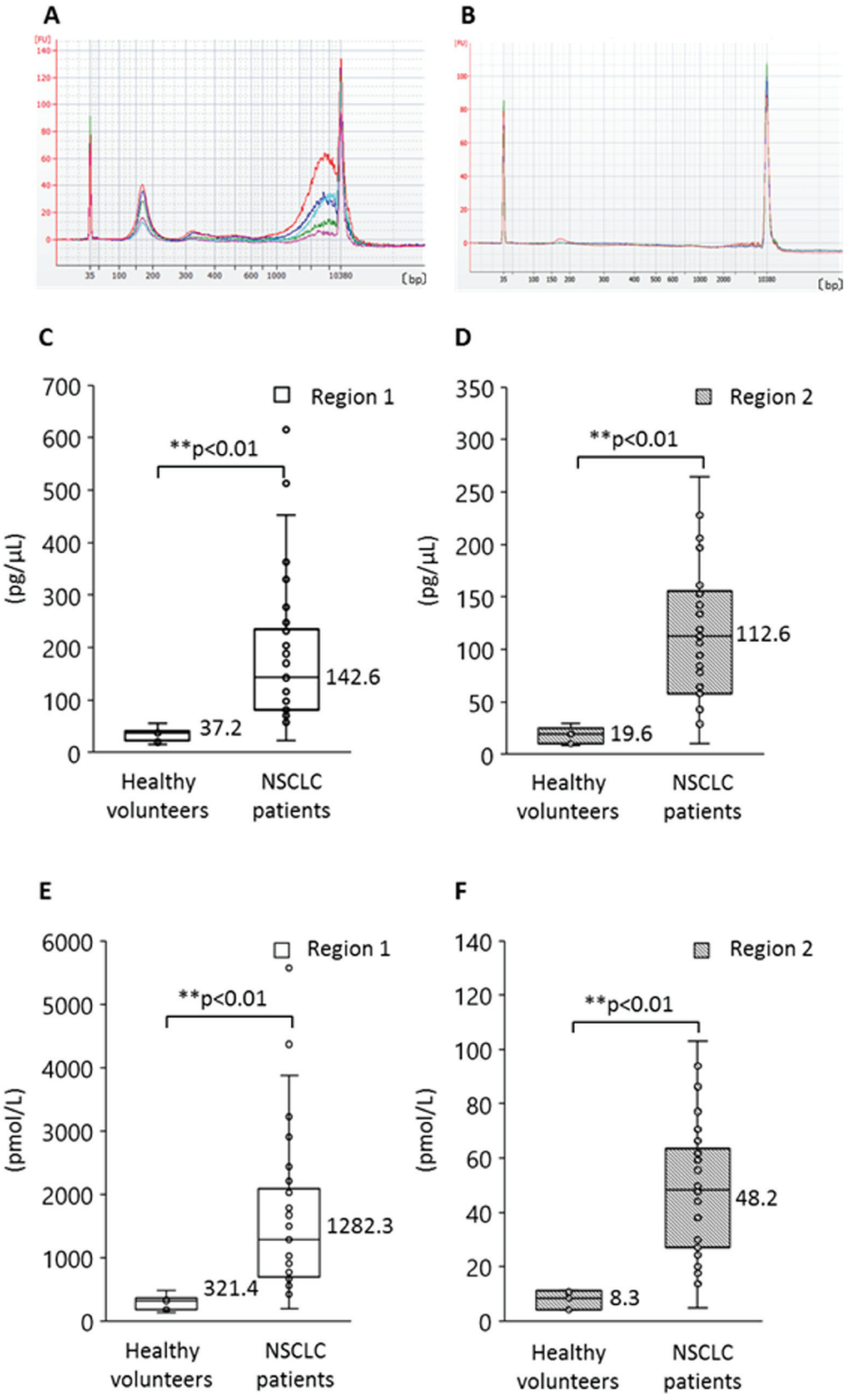

Supplementary Figure 3: Amounts of DNA in regions 1 and 2 in patients with NSCLC and healthy volunteers. The representative size-distribution pattern of plasma DNA isolated using 1000-A is shown for NSCLC patients (**A**) and healthy volunteers (**B**). The concentration (**C**, **D**) and molality (**E**, **F**) of regions 1 and 2 are shown. These were calculated with an Agilent 2100 Bioanalyzer equipped with the Expert 2100 software. Results were obtained with the Mann–Whitney *U* test.

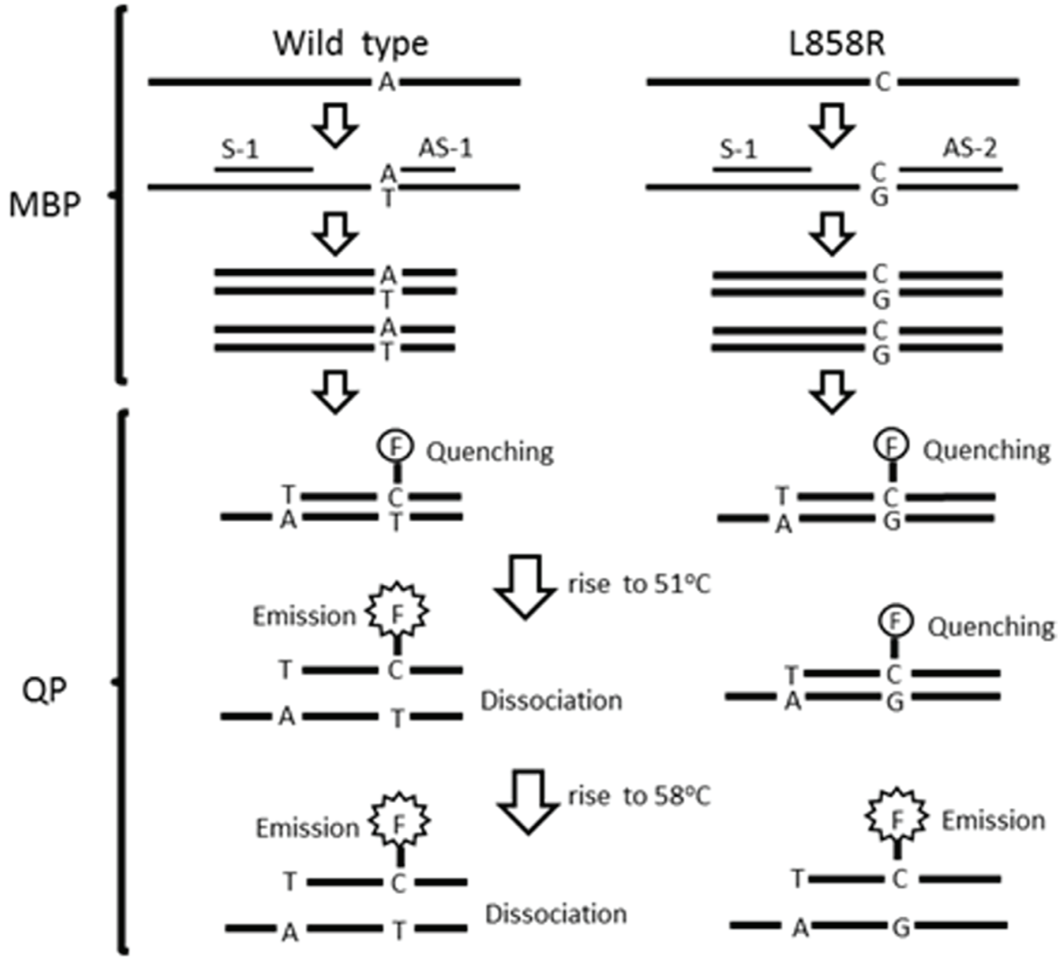

Supplementary Figure 4: Principle of the MBP-QP method. This fully automated, highly sensitive mutation detection system comprises 2 steps. At the MBP step, the targeted gene including point mutation is amplified by two different primers. The primer for mutants is designed to be much longer than that for WT, which allows us to efficiently amplify the mutant allele. At the QP step, a TAMRA conjugated guanine quenched probe, which is designed to match perfectly with mutant amplicons, dissociates from the WT amplicon at lower temperature. Thus, the sample with mutant allele shows two peaks of fluorescence in the measurement pattern.

Supplementary Table 1: Patient characteristics in this study

		Tissue L858R (+)	Tissue L858R (–)
Number of patients		41	20
Sex (% of female)		80%	35%
Never smoker		66%	30%
Stage IV	M1a	29%	25%
	M1b	54%	65%
Recurrence		17%	10%
